# Topology Optimization of the Clutch Lever Manufactured by Additive Manufacturing

**DOI:** 10.3390/ma16093510

**Published:** 2023-05-03

**Authors:** Aleksandra Mikulikova, Jakub Mesicek, Jan Karger, Jiri Hajnys, Quoc-Phu Ma, Ales Sliva, Jakub Smiraus, David Srnicek, Samuel Cienciala, Marek Pagac

**Affiliations:** 1 Department of Fundamentals of Machinery Design, Faculty of Mechanical Engineering, Silesian University of Technology, 44-100 Gliwice, Poland; 2 Department of Machining, Assembly and Engineering Metrology, Faculty of Mechanical Engineering, VSB-Technical University of Ostrava, 70800 Ostrava, Czech Republic; 3 Institute of Transport, Faculty of Mechanical Engineering, VSB-Technical University of Ostrava, 17. Listo-padu 15/2172, 70800 Ostrava, Czech Republic

**Keywords:** topology optimization, additive manufacturing, 3D printing, selective laser melting, SLM, AlSi10Mg, finite element method, FEM

## Abstract

This article aims to review a redesign approach of a student racing car’s clutch lever component, which was topologically optimized and manufactured by Additive Manufacturing (AM). Finite Element Method (FEM) analysis was conducted before and after a Topology Optimization (TO) process in order to achieve equivalent stiffness and the desired safety factor for the optimized part. The redesigned clutch lever was manufactured by using AM–Selective Laser Melting (SLM) and printed from powdered aluminum alloy AlSi10Mg. The final evaluation of the study deals with the experimental test and comparison of the redesigned clutch lever with the existing part which was used in the previous racing car. Using TO as a main redesign tool and AM brought significant changes to the optimized part, especially the following: reduced mass of the component (10%), increased stiffness, kept safety factor above the 3.0 value and ensured the more aesthetic design and a good surface quality. Moreover, using TO and AM gave the opportunity to consolidate multi-part assembly into a single component manufactured by one manufacturing process that reduced the production time. The experimental results justified the simulation results and proved that even though the applied load was almost 1.5× higher than the assumed one, the maximum von Mises stress on the component was still below the yield limit of 220 MPa.

## 1. Introduction

Computer-Aided Design (CAD) software has revolutionized the designing process and enables engineers to produce drawings at great speed and accuracy. Nowadays, engineers need access to a number of tools that improve their work in the designing process. From CAD tools, through 3D printers, to virtual reality and artificial intelligence, today’s technology extends the possibilities of engineering. One of the examples of modern methods of engineering design is the Topology Optimization (TO) process, which, based on mathematical algorithms, significantly facilitates the work of an engineer by proposing new form solutions in the context of engineering structures. TO is a type of computational method that allows to obtain the optimal distribution of the material in a given design space for a specific set of constraints (e.g., loads, boundary conditions) [1,2]. TO has a wide range of applications in multiple disciplines such as automotive [3,4], aerospace [5,6], architecture [7,8], biomedical engineering [9,10] and engineering structures design [11,12,13,14]. The use of TO in the designing process enables a significant reduction in the mass of the designed product while maintaining the desired strength properties and stiffness. The outcome of the TO process is often a new geometric form of a given product, sometimes having a non-technical original shape. Earlier developments of TO considered conventional manufacturing technologies (such as milling, turning or casting) that have limitations in producing complex geometries. Three-dimensional printing (3D printing) brought new opportunities to manufacture complex geometries with undercuts or cavities and to reduce the amount of waste material [15]. Combining these two methods, the TO process and 3D printing give limitless possibilities to the engineers and create a new amount of possible solutions in the designing and manufacturing process [16].

**Figure 1:**
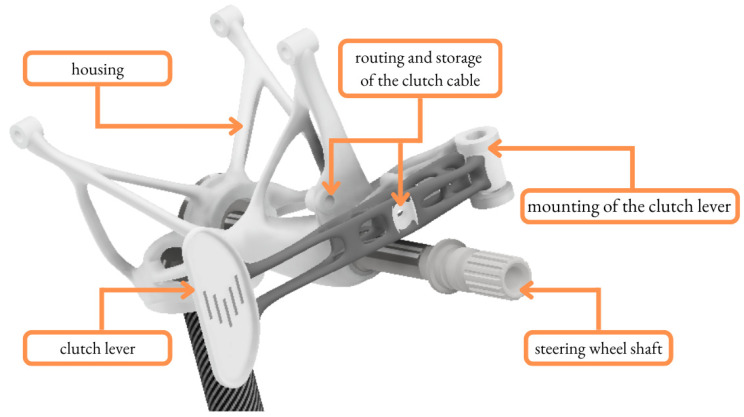
Clutch lever in virtual assembly.

This study was an outcome of the collaboration between Additive Manufacturing (AM) laboratory ProtoLab [17] and one of the Czech student racing teams, who participated in an international engineering design competition where student teams from around the world design, build, test and race a small-scale racing car following the rules and requirements [18]. The trend in student teams is to redesign parts of the racing car to minimize the mass and to maximize the stiffness of the construction [19,20]. This study aims to investigate the role of TO and 3D printing in the redesigning process of the existing part, the clutch lever (Figure 1).

The main purpose of the research was to review the TO process of the clutch lever which is manufactured by AM as a single component. Furthermore, the goal was to reduce the mass of the clutch lever over the preexisting design and to ensure its good surface quality. 

This study has a significant impact for the designing process paradigm for student racing car components and also for other fields of industry such as automotive, aircraft, etc. The novelty of the research refers to applying the TO in practice. The topologically optimized part was tested virtually and experimentally and then implemented into the steering system of the racing car. Moreover, this study deals with implementing TO into the designing process as a regular step of the workflow, which creates a new methodology and minimizes the final mass of the component and enables the manufacture of the product by AM. The set of procedures used in that redesigning process (such as TO steps, Finite Element Method (FEM), preparing the CAD model for AM) was analyzed and could be a standard and universal research plan for future studies.

The paper is organized as follows. Section 1 (Introduction) includes the description of the problem, the purpose of the study and also the description of the study background. Section 2 (Materials and Methods) reviews the material, equipment, techniques, etc. The results and main findings of the study are described in Section 3 (Results). Section 4 (Discussion and conclusions) indicates the main conclusions and interpretations. The last section (References) includes the literature references.

## 2. Materials and Methods

**Figure 2:**
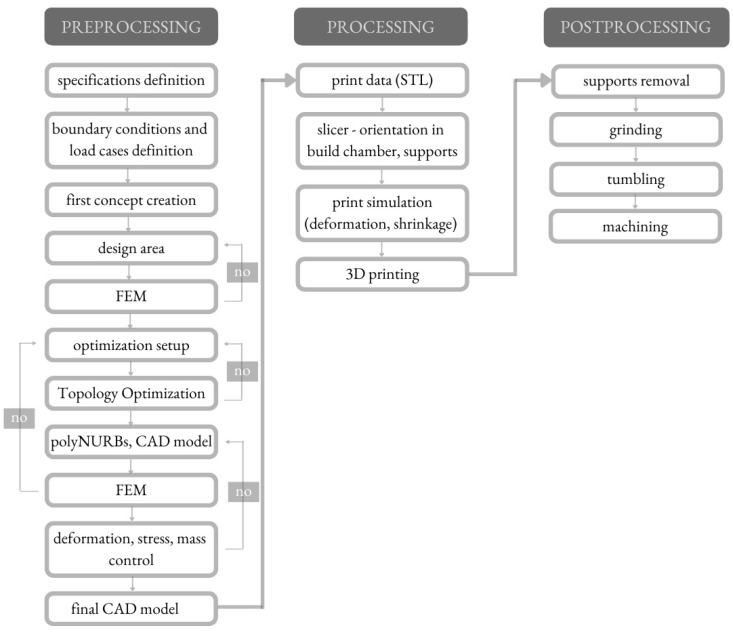
Designing process workflow with TO procedure [21].

The whole redesigning process of the clutch lever was carried out according to the scheme shown in Figure 2.

The process contained a series of procedures including preprocessing operations (CAD model designing, TO, FEM validation), processing operations (preparation of the model for AM, 3D printing) and postprocessing operations. Preprocessing operations contain all the operations connected with CAD model designing. After identifying the need, preprocessing operations started by defining the specifications and constraints (boundary conditions, load cases). Then, the CAD model of the clutch lever was created. The entire component was divided into parts (preserved geometry and design area). The next step of the designing process was to conduct the FEM analysis of the designed component under the load cases. In the case of the FEM analysis outcome not being sufficient, it is necessary to repeat the previous step. When the FEM analysis outcomes were correct, then the TO process was started. The outcome of the TO process was a virtual model, which needed to be converted into the solid model by using polyNURBS. The solid model needed to be FEM-analyzed again to determine how critical factors might affect the entire structure and if failures might occur. When the FEM analysis outcome was correct, the next step was to prepare the final CAD model and to export it to print data. This step was the beginning of the processing operations, which contain the whole process of preparation of the model for AM (among others, slicing the model, print simulating, etc.) and the final 3D printing of the clutch lever from powdered metal AlSi10Mg by using the Selective Laser Melting (SLM) process. Postprocessing operations contain several operations such as support removal, grinding, tumbling and machining. These operations were conducted to prepare the part for the intended form (especially dimensions) and function. After the whole redesigning process, the experimental study was carried out. 

### 2.1. Material

**Figure 3:**
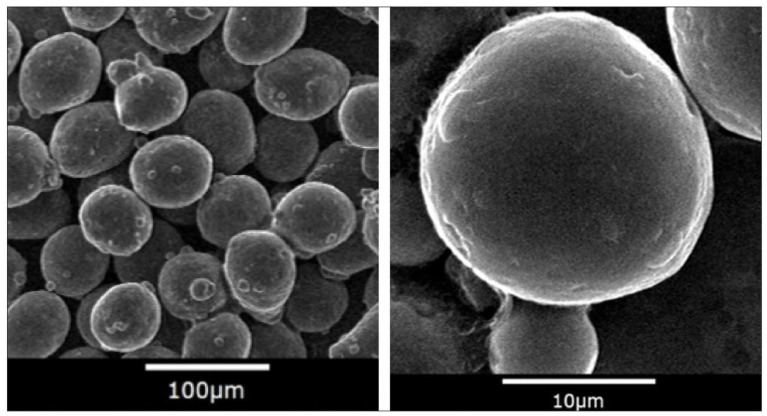
Spherical AlSi10Mg powder [23].

One of the first steps in the TO process is to define the material of the parts which will be optimized. The clutch lever is planned to be manufactured by Selective Laser Melting (SLM) from powdered metal. In this study, the mass of the final component is a deciding factor, so aluminum alloys were chosen. Aluminum alloys, along with their low density, provide good electrical conductivity as well as mechanical and thermal properties [22]. There are several types of aluminum alloy powders available on the market and one of the most popular used in SLM is aluminum silicon magnesium (AlSi10Mg) alloy powder, which was chosen for this study (Figure 3). 

AlSi10Mg powder is a kind of aluminum-based alloy powder with aluminum as the main component and a small amount of silicon, magnesium, iron and other elements. Among them, the silicon content is 9.0–11.0%, and the magnesium content is 0.25–0.45%. The iron content is smaller than 0.25%. The detailed chemical composition is shown in Table 1. 

**Figure 4:**
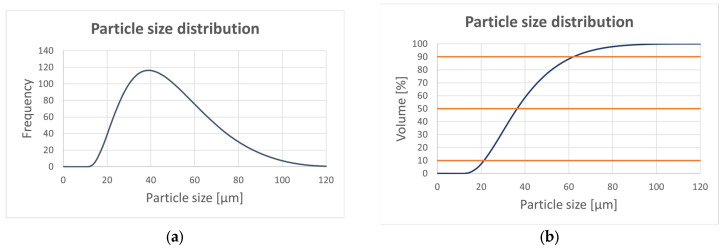
Particle size distribution (**a**) in frequency and (**b**) in volume (%).

The microstructure of the AlSi10Mg samples was examined by scanning electron microscopy (SEM) in the backscattered electron (BSE) and secondary electron (SE) modes using a Quanta 450 FEG microscope equipped with an energy dispersive X-ray spectrometer (EDS). The particle size distribution can be observed in Figure 4 with the data listed in Table 2.

**Figure 5:**
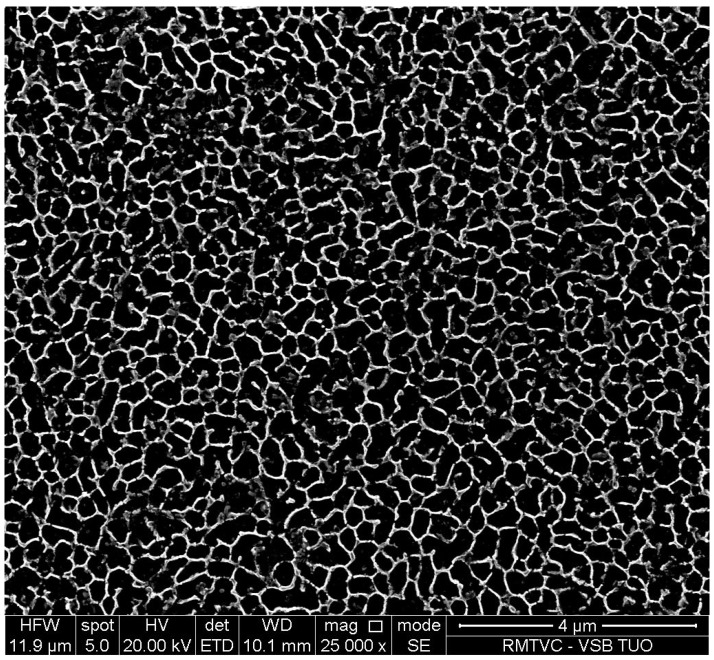
Microstructure of as-built AlSi10Mg sample produced by SLM.

AlSi10Mg is an aluminum alloy with good hardness, strength and dynamic toughness which is traditionally used as a casting alloy. Powder made from AlSi10Mg is widely available in the AM market, due to the high corrosion resistance, low density and high mechanical strength of the final components [24,25,26,27,28]. Additionally, it is relatively easy to print in comparison with other metallic materials. During the SLM process, the residual stress occurs in the components and it affects the final shape distortion. According to the previous study [29], the authors made the appropriate printing adjustments (mainly usage of supports or part orientation and design changes, etc.) to minimize the residual stress. The AlSi10Mg also has the ability to improve mechanical properties after the heat treatment [30,31]. Applying artificial aging in the postprocessing could cause the increase in hardness, Young’s modulus and strength and also a decrease in residual stress [32]. In Figure 5, we can observe a typical microstructure of AlSi10Mg components manufactured by SLM. The dense mesh covering the dark material is the Si network, which is the main strengthening mechanism of the material if it is heat-treated appropriately [32]. Specifically, after 6 h of heat treatment at 170 °C, the yield strength (Sy) is increased from 279 MPa to 310 MPa and the ultimate strength (Su) from 409 MPa to 448 MPa. Together with strengths, the hardness is significantly improved from 127 HB to 143 HB. This is caused by the precipitation of Si particles while they coarsen inside the material cells [32].

The basic parameters of the powdered AlSi10Mg and parameters obtained after heat treatment are shown in the following Table 3.

After defining the material, it is necessary to assign this material to the whole optimized part. The small library of common materials is included in Altair Inspire, but the software has the ability to create new materials. A new material is defined by few parameters that characterize basic linear properties such as Young’s modulus (E), density and yield stress. The coefficient of thermal expansion (α) and the thermal conductivity (λ) are used only in cases where temperatures have been added to a load case.

### 2.2. CAD Model

**Figure 6:**
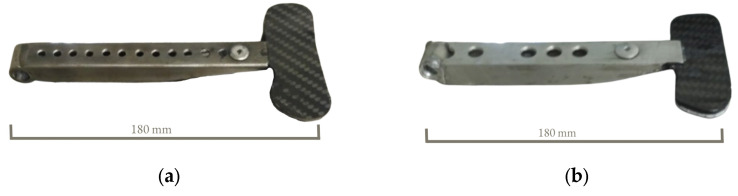
Already existing clutch levers: (**a**) the clutch lever made from steel and carbon fiber plate; (**b**) the clutch lever made from aluminum alloy and carbon fiber plate.

The initial design of the clutch lever was based on two already existing models of clutch levers which were used in the last editions of student racing cars. These previous versions of clutch levers were multi-part assemblies of steel and carbon fiber parts (Figure 6a) or aluminum alloy and carbon fiber parts (Figure 6b), combined by riveting and welding. The steel–carbon fiber clutch lever weighs about 90 g and the aluminum alloy–carbon fiber clutch lever weighs 52 g.

Based on the already existing clutch levers, the following targets were defined:

- to reduce the mass while maintaining acceptable strength and stiffness using TO;
- to keep the main dimensions;
- to design a clutch lever as a single component;
- to ensure attractive design;
- to obtain smooth surface without burrs;
- to keep minimum safety factor above 3;
- to manufacture the component by AM.

**Figure 7:**
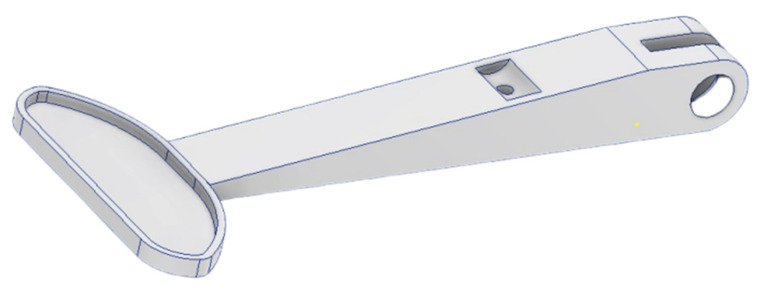
Oversized initial CAD model.

The initial design of the clutch lever (Figure 7) was appropriately simplified and oversized to provide the area for the distribution of material during the TO.

**Figure 8:**
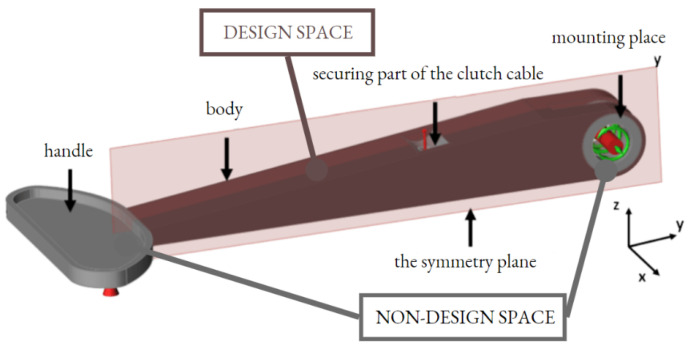
Symmetry boundaries of a design area.

The main dimensions of the model are based on the existing models, due to the limitation of the built-in space with the instrument panel, the steering box and the steering wheel. The finished CAD model was converted into the STEP format, which Altair Inspire software was able to read and edit. The initial model of the clutch lever was divided into three components (handle, body, lever mounting) for the optimization process and because the lever body would be optimized (design space), so it was necessary to set the symmetry plane for the symmetrical generation of the body shape (Figure 8). Before the optimization starts, it is important to specify the places that must not be optimized (non-design space). In this case, areas which could not be optimized were the handle part, the mounting place and also the part which secures the clutch cable.

Using the symmetry boundary during TO shortened the time of the process and ensured better manufacturing parameters while printing the symmetrical part.

### 2.3. Load Cases

**Figure 9:**
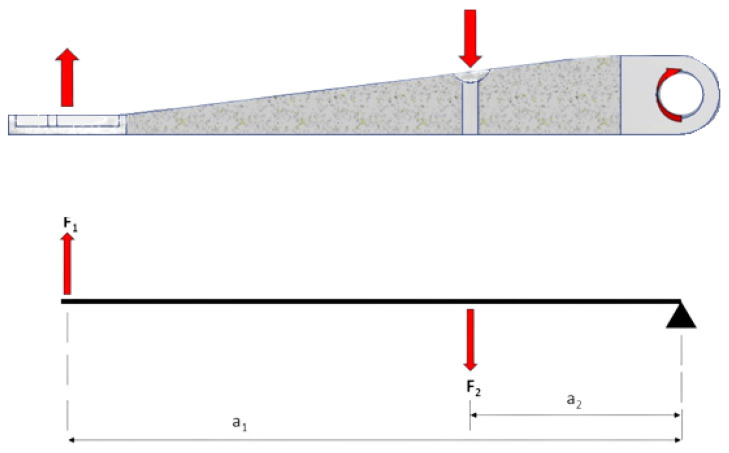
Load case.

The next step of the TO process was determining the forces, reactions, bonds (bonding contacts between the parts) and all other information, which are needed to solve the optimization process (Figure 9). The action force is exerted by the driver on the handle of the clutch lever, when pressing the lever towards the steering wheel. When the clutch handle is pressed, a reaction force acts in the opposite direction. The mount of the lever is defined as a rotational coupling with degrees of freedom both rotating about the X-axis in both directions and sliding along the X-axis.

The experimentally determined (by using the existing clutch lever) force F_1_ value is approximately 125 N at a distance a_1_. To calculate the reaction force F_2_, the following equation was used:

F_1_ ∙ a_1_ = F_2_ ∙ a_2_, (1)

where

F_1_–pressure force induced by the driver (N)

F_2_–reaction force induced by clutch cable (N)

a_1_–distance between force F_2_ and the axis of rotation (mm)

a_2_–distance between force F_1_ and the axis of rotation (mm)

The reaction force F_2_ is calculated with F_1_ = 125 N, a_1_ = 155 mm, a_2_ = 53 mm.

F_2_ = 365.6 N ≅ 366 N. (2)

The reaction force exerted by the clutch cable is therefore 366 N.

### 2.4. Topology Optimization

Altair Inspire software uses OptiStruct structural solver in the background, and thus the TO is based on the Solid Isotropic Material with Penalty (SIMP) method. By discretizing the domain into a finite element mesh, OptiStruct calculates material properties for each element. The OptiStruct algorithm alters the material distribution to optimize the user-defined objective under given constraints. In the SIMP method, a pseudo material density is the design variable, and hence it is often called the density method as well. The material density varies continuously between 0 (void state) and 1 (solid state). The SIMP method applies a power law penalization for the stiffness–density relationship in order to push density toward 0/1 (void/solid) distribution to any 2D and 3D elements [34]:

Κ(ρ) = ρ *^p^* ∙ Κ. (3)

where Κ is the penalized stiffness matrix of an element, Κ is the real stiffness matrix of an element, ρ is the density and *^p^* is the penalization factor (always greater than 1).

The TO process has few parameters to setup before optimization begins. The target for the TO process was set up to maximize the stiffness of the component when reducing its mass with a final mass percentage target: 15% of the existing lever’s body. “Thickness constraints” are thickness restrictions that are used to analyze the maxima and minima of the range of elements, where the maximum value is at least twice as large as the minimum. It can be set manually, to speed up the calculation, but in this case, it was automatically determined as the lowest possible thickness for a more accurate calculation even at the cost of a more time-consuming processing step. All parameters are shown in Table 4.

The mass target specifies the amount of material to keep. The initial mass target was set as a percentage (15%) of the total volume of the design space. Element size dictates the quality of the optimization result. In general, the smaller the element size, the more accurate the result but the longer time required to perform a computational process. Depending on the computational power and limited time for this study, element size was set at the value of 4.3 mm. Based on the FEM analysis, the set value is sufficient for this type of component. The outcome of the TO process is a rough theoretical model with sharp edges, which can be smoothed and then worked on using the “PolyNURBS” function.

**Figure 10:**
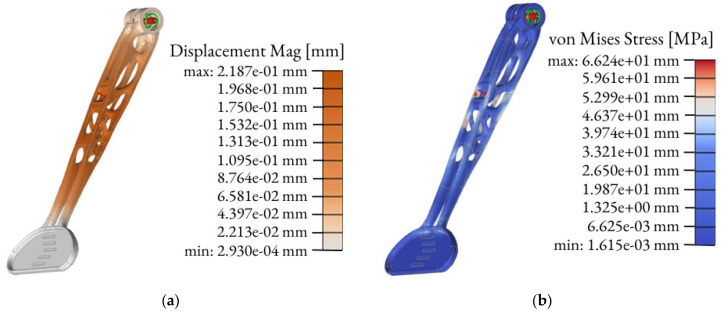
Engineering analysis of the outcomes: displacement (**a**) and von Mises Stress (**b**).

The solid model after TO should be verified by FEM analysis. The topologically optimized clutch lever was FEM-analyzed according to the load cases assumed in Section 2.3. The result of this study is shown in Figure 10.

**Figure 11:**
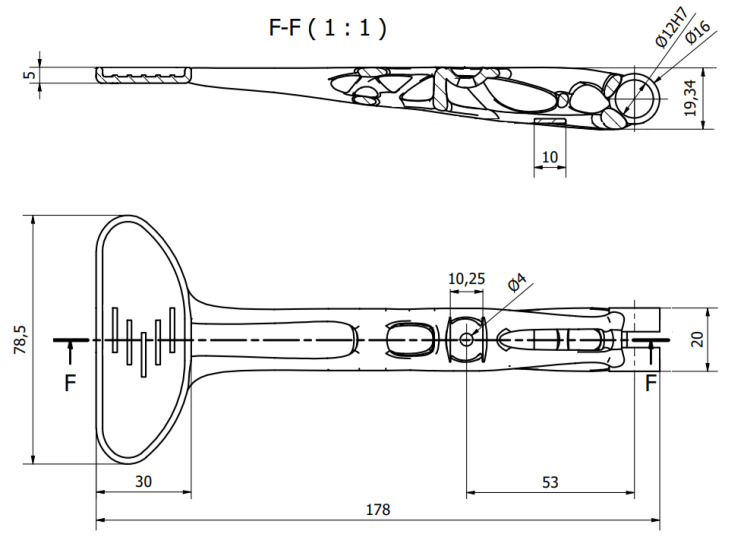
Main dimensions of the topologically optimized clutch in mm.

The linear static analysis is the common type of FEM analysis which is often used for the first estimate of the stresses and displacements of a model [35]. The linear static analysis in Altair Inspire software automatically runs few types of analyses for each load case. The outcome of the FEM analyses include displacement, factor of safety, percent of yield, tension and compression, maximum shear stress, von Mises stress plus major and minor principal stress. The final model of the topologically optimized clutch with its main dimensions is shown in Figure 11.

### 2.5. Technical Verification

Table 5 shows the overview and comparison of the clutch lever models including initial models used for the student racing car (the combination of carbon fiber–steel and carbon fiber–aluminum alloy Al6061 parts) and their parameters. The parameters are the outcomes of the FEM analysis of CAD models tested with the same load cases mentioned in Section 2.3.

**Figure 12:**
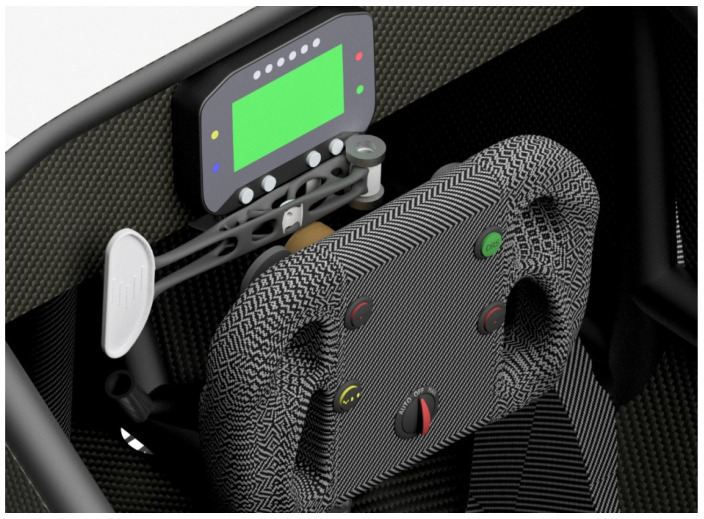
Visualization of the redesigned clutch lever mounted to the steering system.

To verify the outcome technically, it was necessary to analyze the virtual assembly of the whole steering system and the optimized clutch lever (Figure 12).

Virtual fitting of the clutch lever into the assembly enables one to check the assembly before the manufacturing, to identify design problems (if there are any) and to assess the assembly visually.

### 2.6. Additive Manufacturing

**Figure 13:**
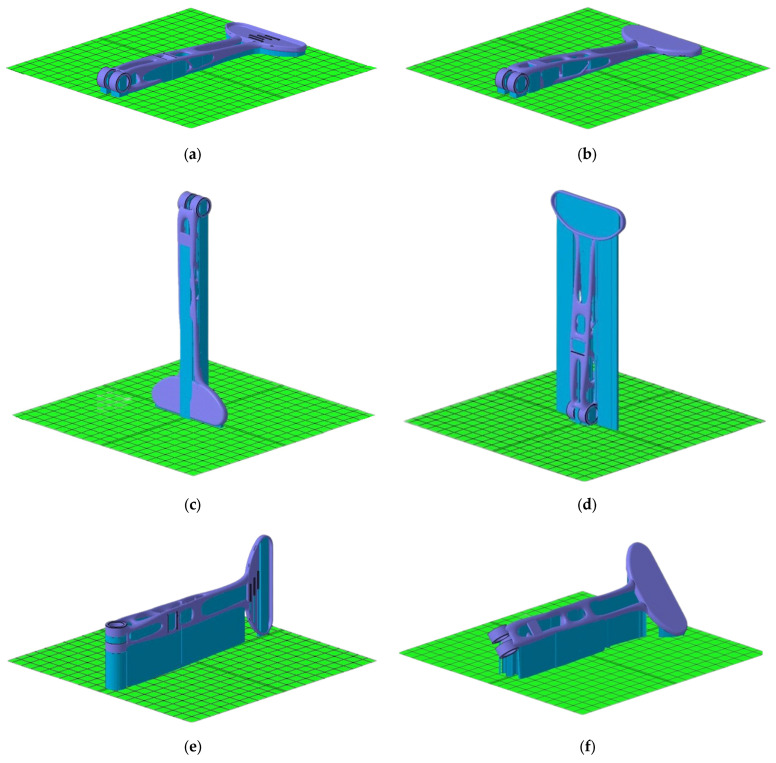

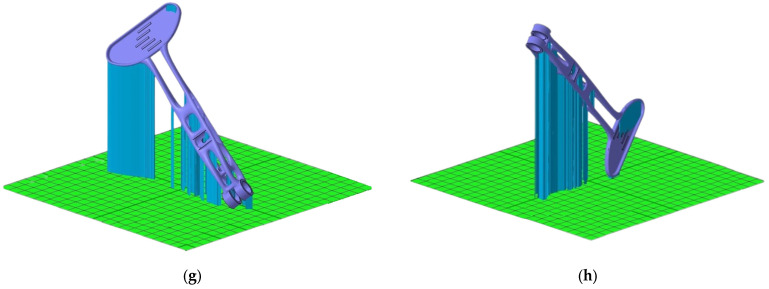
Showing 3D printing orientation using Autodesk Netfabb software. Taking the baseplate as the reference, we have: (**a**) parallel orientation 1, (**b**) parallel orientation 2, (**c**) perpendicular orientation by longitudinal axis 1, (**d**) perpendicular orientation by longitudinal axis 2, (**e**) perpendicular orientation by side 1, (**f**) perpendicular orientation by side 2, (**g**) inclined orientation 1, and (**h**) inclined orientation 2.

The final redesigned model of the clutch lever was manufactured by using SLM. SLM is one of the most commonly used Laser Additive Manufacturing (LAM) methods which has the potential of manufacturing a part without postprocessing [36,37]. The SLM process is appropriate for fabricating high-density parts with a complicated geometry by melting metallic powders via a high-power-density laser. The working principle of the SLM process initiates by applying a thin powder layer into the building platform, then the applied powder is wholly melted by the thermal energy induced by one or several laser beams [38]. Due to its mechanical property and lightweight characteristics, the demand for utilization of the SLM process has swiftly increased in several industries, including aerospace [39], automotive [40], biomedical [41] and other high-tech areas. The clutch lever was printed using Renishaw AM500E. The RenAM 500 series features a build volume of 250 mm × 250 mm × 350 mm, which is sufficient to print the clutch lever as a single component. Print data were prepared with the Autodesk NetFabb software (Figure 13). Netfabb is an AM software tool used for preparing, cleaning, slicing, toolpathing and even simulating AM [42]. The orientation of the piece has an influence on several factors such as the surface finish [43], the “resolution” of the piece—its level of detail—and the heat resistance. The finish of the printed part will depend mainly on two factors: the geometry of the part and the number of supports required. It is important to correctly rotate and arrange parts on the build platform for optimized use of space and printing time. Using a NetFabb, a total of 8 possible orientations were iterated to find the appropriate print location on the build platform.

Table 6 evaluates and compares parameters such as the area content of the supporting material, the volume of the supporting material, the size of the outbox volume, the height of the model position and the height of the center of gravity of the model in that orientation. This evaluation is presented numerically and in colors (where green—recommended; yellow—sufficient; red—not recommended), and the orientations of the component are arranged in ascending order from recommended (optimal) to inappropriate orientations.

Some of the shown orientations would have been more time-consuming and created more material waste during manufacturing. The amount and the volume of the supporting material have a significant influence on the printing process and the final result. Supporting material provides significant heat dissipation and minimizes deformations when the part is printed. Supporting material is necessary to print the lever mounting holes to avoid their deformation or collapse. According to Table 4, the number 1 and number 2 positions were chosen as the best orientations for the clutch lever. Both of them have a significantly low volume of the supporting material. These positions have the lowest height of the center of gravity and the lowest height of the model position (very close to the building platform). The overall height results in a significant reduction in printing time. Positions numbered 3, 4, 6 and 7 are quite unsuitable due to the high overall height, as a large amount of material would be used to build their supports and at the same time, their printing time would be high. Besides the un-optimal use of building space, material and time, for vertically positioned components, there is also the risk of insufficient cooling, which could lead to high distortion of the part or, even more severe, the print being aborted.

**Figure 14:**
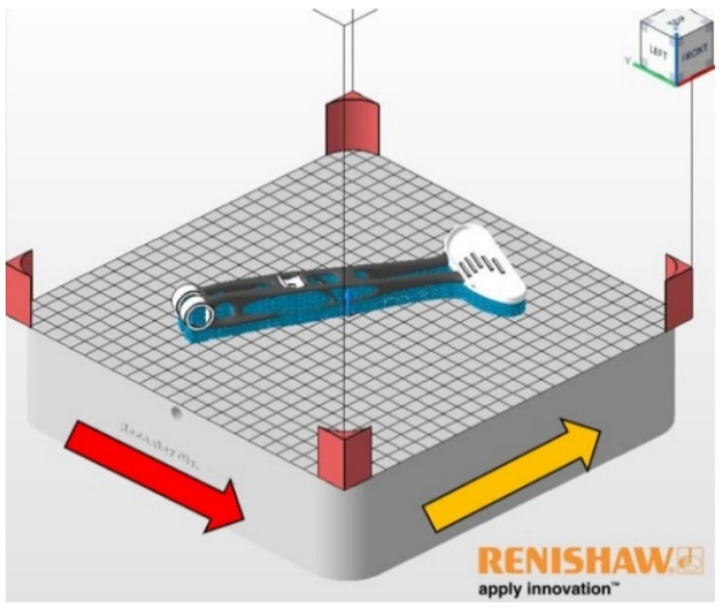
The final orientation of the lever in the build chamber.

The lever in the press chamber was oriented following the number 1 position and rotated 28° in the negative direction around the “Z” axis (Figure 14).

With this rotation, we achieve more suitable application capabilities of the recoater. The red arrow shows the direction of the powder application in the printing chamber. The yellow arrow indicates gas flow of argon. The lever model is located in the press chamber 5 mm above the build plate for better support creation.

### 2.7. Mechanical Analysis of the Printing Process

The mechanical analysis of the printing process was conducted using the Simufact Additive software. Simufact Additive is a powerful and scalable process simulation environment for the optimization of Metal Additive Manufacturing processes [44]. Simufact Additive features help to solve the main issues of metal 3D printing such as reducing distortion, minimizing residual stress to avoid failure, optimizing build-orientation, optimizing support structures, etc. [45]. Distortion is an unavoidable result of heating a small amount of material to its melting point on an otherwise much colder entity. Distortion is unavoidable, but it can be mitigated by geometry compensation. Compensation is a way of changing the part geometry by anticipating the distortion that will occur during fabrication, so that the part distorts into the desired final shape [46,47,48].

**Figure 15:**
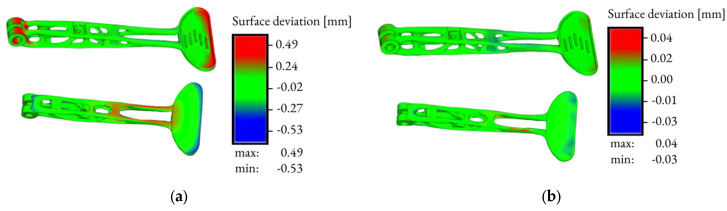
Mechanical analysis of the printing process: final distortion without compensation (**a**) and with compensation (**b**).

Figure 15 presents images of the final distortion without compensation and with compensation.

The software allows to calculate the final distortion and to pre-define the compensation, so that during and after printing, the dimensions are as accurate as possible and the part is not deformed. The simulation was performed without software-machine calibration, so minor deviations may occur. The thickness of the printed layer was set to 30 µm. Figure 13a depicts the final distortion without compensation. The maximum deviation shown in red reached a value of 0.49 mm. The minimum deviation value without compensation reached a value of −0.53 mm. These values evidence that the lever will be bent after printing, so it was necessary to create a compensation.

The next step was to run the simulation to automatically calculate what the compensated geometry should be, to obtain the first print right. The allowable distortion value was pre-defined to be 0.05 mm maximum and −0.05 mm minimum in the software. After evaluating the compensation, the simulation values of the maximum deviation came out to 0.04 mm. The minimum deviation value was −0.03 mm. Performing the mechanical simulation of the entire build process helped to predict distortions and defects before printing a part.

### 2.8. Postprocessing

Postprocessing techniques are certain mechanical or thermal operations performed on additively manufactured components to ensure a proper reliability. The first postprocessing operation was to separate the lever from the base plate by using a band saw PEGAS 360 × 500 SHI-LR. Furthermore, the postprocessing consisted of the mechanical removal of supports and their scraps, using extended pliers, side slitting pliers and various metal files. Then, wet tumbling was conducted on the OTEC CF1 × 32 EL device in combination with ceramic bodies FATHER DZS 10/10 for two hours.

There is also room for a heat treatment operation at the beginning of postprocessing. Heat treatment alters the properties of a metal without changing its shape. According to the previous studies [29,32] as aforementioned in Section 2.2., the heat treatment conducted by using an artificial aging process (which involves elevating the temperature of an alloy to 170 °C and annealing the component in this temperature for 6 h) improves mechanical properties and decreases residual stress. The reason why the heat treatment was not implemented in this research was mainly because of different machine specifications, different material manufacturer and other printing parameters, etc., than in conducted research. The possibility of implementing the heat treatment operation after the SLM process at the Renishaw machine is a scope for a future work.

### 2.9. Experimental Study

**Figure 16:**
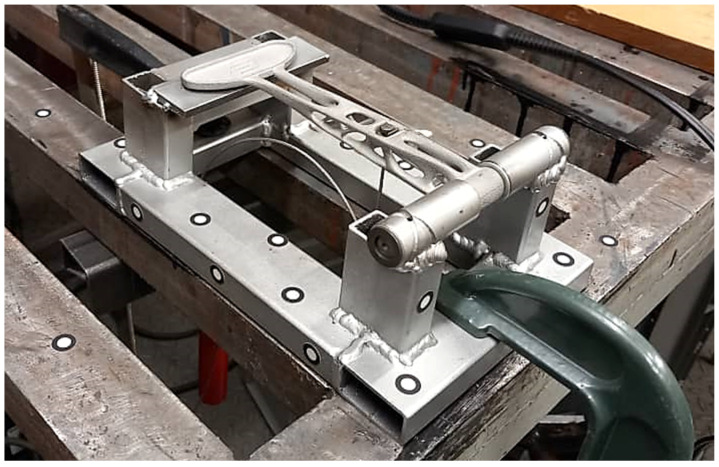
Clutch lever fixed at the position of the pin connection.

**Figure 17:**
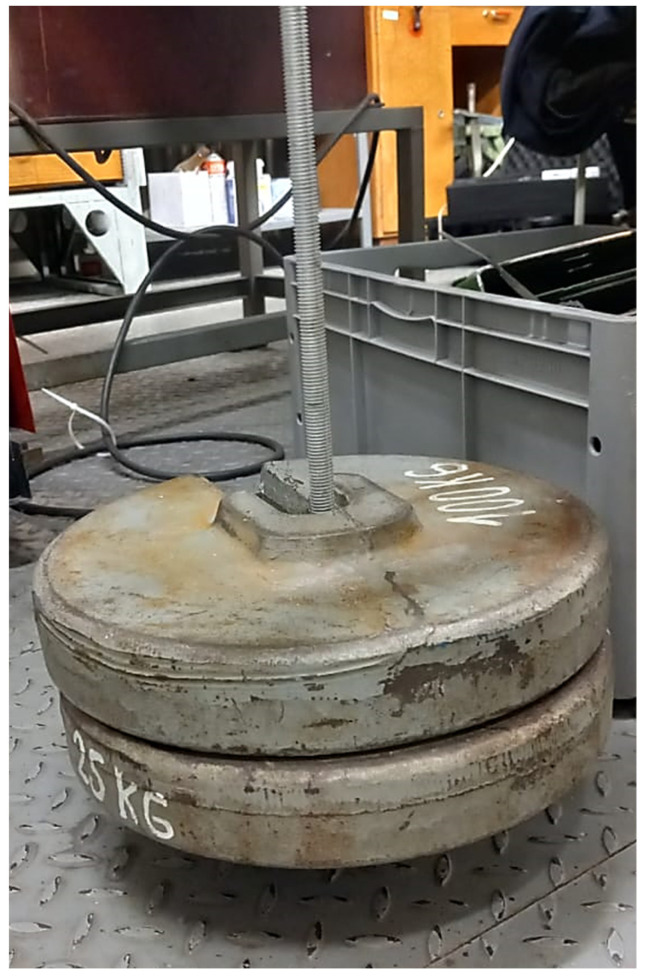
The force of 522.5 N applied to the clutch lever.

For experimental study, the clutch was subjected first to FEM analysis and then to physical tests. Marc Mentat 2020 Feature Pack 1 was used for FEM analysis. The clutch was meshed with 65,616 tetrahedral elements and fixed at the position of the pin connection (only rotation around the Z axis was allowed). Its other end was in contact with a solid cube that represents the testing stand. The simulation was linear with the material properties as in Table 3 (material parameters). A force of 522.5 N was applied at the position where the cable is mounted in reality. This corresponds to the test setup in Figure 16 and Figure 17.

The force of 522.5 N was calculated from two weights of 25 kg and a hanger of 2.25 kg. During the test, the surface deviation was captured by 3D-scanning the shape of the clutch on the stand before and after the load was applied. Creaform Handyscan (Creaform Inc.) was used for scanning purposes.

**Figure 18:**
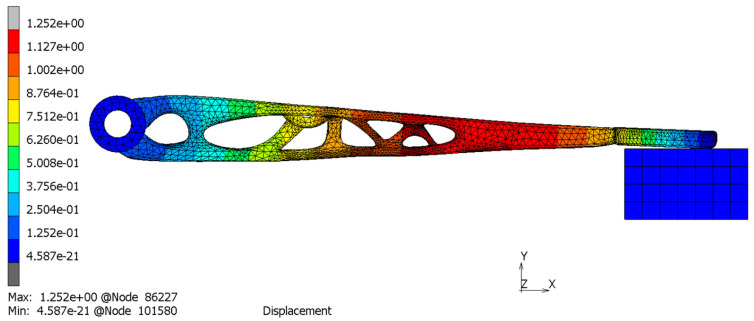
Displacement analysis.

**Figure 19:**
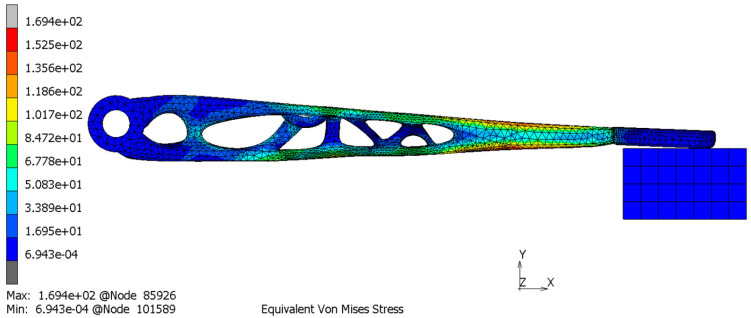
Equivalent von Mises stress analysis.

**Figure 20:**
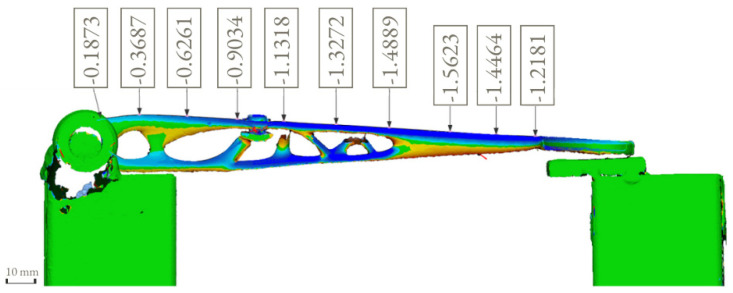
Surface deviation analysis during the test.

The displacement, von Mises stress and surface deviation results are shown in Figure 18, Figure 19 and Figure 20, respectively.

It can be observed from the simulation that the maximum von Mises stress is at the bottom layer of the clutch with a maximum stress value of 169 MPa and a maximum displacement of 1.25 mm. The position and the displacement results are comparable with the 3D scanning results that show the maximum displacement of 1.56 mm. The distortion detected by the 3D scanning (1.56 mm) was slightly higher than the simulation results (1.25 mm) because, in the simulation, we considered the ideal geometry of the component, the stand for testing and other idealized constraints for the load.

## 3. Results

Based on the simulation results (Table 5), the final optimized clutch lever upon comparison with the preexisting one (made of carbon fiber) gave significant differences, especially for mass and the number of parts. The preexisting clutch lever weighs 52 g while the optimized lever weighs 10% less—47 g. When comparing the safety factors of both of the components, it can be noticed that even though the value is smaller for an optimized clutch lever (6 to 3.3), the result is still sufficient. The maximal deflection is relatively larger than it was in the aluminum–carbon fiber clutch lever, and so are the maximal von Misses stress, but both of these values are acceptable.

The experimental result confirmed the simulation results and justified the reason to apply TO into the designing process of the clutch lever. Remarkably, even though the load value of 522.5 N applied to the clutch lever was almost 1.5× higher than the calculated value of 366 N (see: Equation (2)), the maximum stress of the analyzed part was still below the yield limit of 220 MPa.

Surface deviation was captured by 3D scanning of the clutch lever on the stand before and after the load of 522.5 N was applied, which shows the maximum displacement of 1.56 mm (Figure 20). This result is comparable with the simulation result (Figure 18) that shows the maximum displacement of 1.25 mm. It can also be noticed that the maximum von Mises stress occurs at the bottom layer of the clutch with a maximum stress value of 169 MPa (Figure 19). Because the maximum von Mises stress is under the yield limit of 220 MPa, within which the stress and strain have linear relation, we can approximately calculate the maximum von Mises stress and maximum displacement under 366 N to be 118.7 MPa and 0.88 mm (for FEM) and 1.09 mm (for 3D scanning). These values both satisfy the allowable value of 1.50 mm. Due to the chosen manufacturing method (SLM), there is a significant reduction in the number of components. The multi-part assembly was consolidated into a single component clutch lever with a new original design and smooth surfaces without burrs.

## 4. Discussion and Conclusions

**Figure 21:**
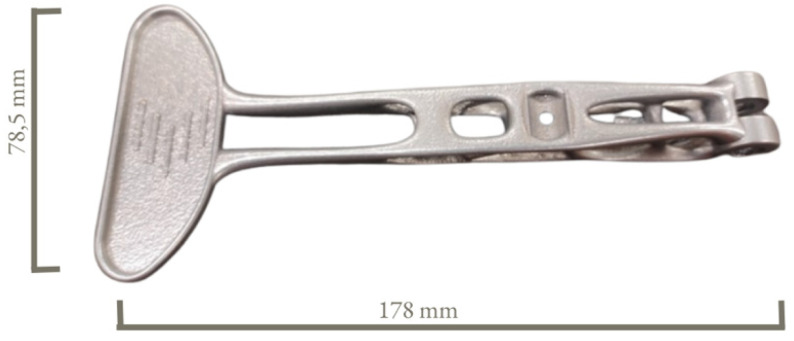
SLM-printed clutch lever, AlSi10Mg.

This article describes a redesigning process of the clutch lever based on the already existing component from the racing cars and applying TO in practice. The whole redesigning process contains a series of procedures including CAD model designing, TO, FEM validation, preparing the model for AM, postprocessing operations, etc. This approach was successfully implemented. The redesigned clutch lever was manufactured by SLM (Figure 21) and it was mounted to the racing car steering system.

The optimized clutch lever meets all the aforementioned requirements in Section 2.2 when it is manufactured as a single component by AM. The topologically optimized clutch lever has acceptable strength with reduced mass with a safety coefficient higher than the required value. Furthermore, the lever meets mounting dimensions, it has a smooth surface without burrs and stands out with a unique design. The strength and other mechanical properties of the clutch lever could be improved by applying the heat treatment operation right after the SLM process. The possibility of implementing the heat treatment operation after the 3D printing at the Renishaw machine is a scope for future work.

The clutch lever is only a part of the entire steering system, and based on the example described in this research, there is a possibility of implementing TO into the redesigning process of other racing car components and to significantly reduce the total weight of the racing car. This study has a significant impact for the designing process paradigm for student racing car components. The set of procedures used in this redesigning process (such as TO steps, FEM, preparing the CAD model for AM) could be a standard and universal research plan for following research studies.

The scope for further research may focus on applying lattice structures to the optimized CAD models. The lattice structure for 3D printing is a repeating or non-repeating three-dimensional assemblage of connected nodes. In its simplest form, multiple lattice nodes are connected to one another by beams. Applying lattice structures to the racing car parts could reduce the mass even more than by using only TO. The contributions of this study are:

1. The TO process and 3D printing give new possibilities to the engineers to create many possible solutions with new original shapes in the designing and manufacturing process;
2. The role of TO and 3D printing is crucial in the redesigning process, which aims to reducing the mass of the component;
3. Using the symmetry boundary during TO made the process shorter;
4. Performing the mechanical simulation of the entire build process helped to predict distortions and defects before printing a part;
5. The redesigned and optimized clutch lever has a reduced mass (10% less), unique design with smooth surfaces without burrs and it is manufactured as a single component in one manufacturing process, which reduced the production time;
6. Simulation results are justified by the experimental analysis, which was conducted by applying a 1.5× higher load than the calculated one;
7. The set of the designing steps including TO could change the traditional paradigm of the designing process and could be applied to any other similar study as standard designing procedure.

## Figures and Tables

**Table 1:** Chemical composition of AlSi10Mg powder [23].

Element	Al	Si	Mg	Fe	N	O	Ti	Zn	Mn	Ni	Cu	Pb	Sn
**Mass (%)**	Balance	9–11	0.25–0.45	<0.25	<0.2	<0.2	<0.15	<0.1	<0.1	<0.05	<0.05	<0.02	<0.02

**Table 2:** Powder analysis (where D [4;3] is a Volume moment mean; D_v_ are the particle size data values which define powders in terms of micron size: they represent 10%, 50% and 90% of the product below the named micron size).

Parameter	D [4;3]	D_v_ (10)	D_v_ (50)	D_v_ (90)
**Particle size (μm)**	39.5	21.3	36.4	62.4

**Table 3:** Material parameters [33].

E (MPa)	Density (g/cm^3^)	Yield Stress (MPa)	α (10^−6^ k^−1^)	λ (W/mK)
69,000	2.68	220	20	190

**Table 4:** Process parameter settings.

Parameter	Set Value
Analysis type	Topology optimization
Objective	Maximize stiffness
Initial mass target	15% of design space volume
Element size	4.3 mm
Geometry constrain	Plane symmetry (longitudinal plane)

**Table 5:** Overview of the results.

Clutch Lever	Mass (g)	Max von Mises (MPa)	Maximal Deflection (mm)	Minimum Safety Coefficient (-)	Number of Components (pcs)
Steel–carbon fiber	90	27.5	0.02	7.4	6
Al6061–carbon fiber	52	41.6	0.09	6.0	6
AlSi10Mg–oversized model (before TO)	111	34.7	0.07	6.3	1
AlSi10Mg (after TO)	47	66	0.22	3.3	1

**Table 6:** Comparison of 8 different part orientation parameters.

No.	Figure	The Area Content of the Supporting Material (cm^2^)	The Volume of the Supporting Material (cm^3^)	The Size of the Outbox (cm^3^)	The Height of the Model Position (mm)	Height of the Center Gravity (mm)
1	14a	42.699	14.363	261.762	18.7	7.3
2	14b	43.448	12.586	274.024	19.6	6.3
3	14c	9.265	19.812	264.762	178.2	84.1
4	14d	9.256	29.049	261.762	178.2	94.1
5	14e	18.816	35.214	261.762	78.5	39.3
6	14f	14.572	31.046	589.297	61.8	30.2
7	14g	4.564	21.218	892.628	150.3	71.3
8	14h	3.822	16.641	517.051	160.2	83.7

**Note**: green—recommended; yellow—sufficient; red—not recommended.

## Data Availability

Not applicable.
